# Supplementary material for: Epsilon-Globin HBE1 Enhances Radiotherapy Resistance by Down-Regulating BCL11A in Colorectal Cancer Cells
Source: Cancers (Basel). 2019 Apr 8;11(4):498. doi: 10.3390/cancers11040498 (PMC6521047; doi:10.3390/cancers11040498)
Supplement: Supplementary file 1 [file cancers-11-00498-s001.pdf]

Supplementary Materials: Expression of epsilon-globin HBE1 enhances radiotherapy resistance via the downregulation of BCL11A in colorectal cancer cells

Sang Yoon Park, Seon-Jin Lee, Hee Jun Cho, Jong-Tae Kim, Hyang Ran Yoon, Kyung Ho Lee, Bo Yeon Kim, Younghee Lee and Hee Gu Lee

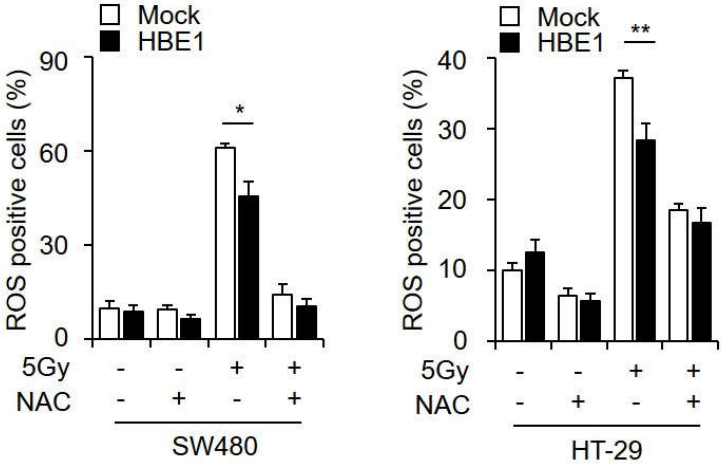

Figure S1. Mock and HBE1 expressing cells were treated with or without NAC and Radiation induced intracellular ROS levels measured by flow cytometry using CM-H2DCFDA at 48 hr after exposure to 5 Gy of irradiation. * $p < 0.05$, ** $p < 0.01$.

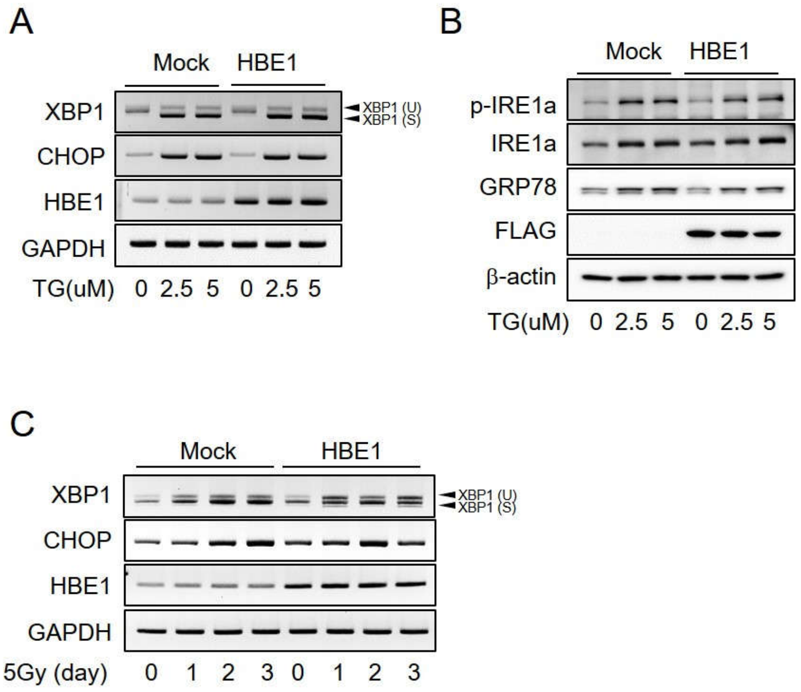

Figure S2. (**A**) Total RNAs were harvested from thapsigargin (TG) treated mock and HBE1 expressing cells and XBP1, CHOP, HBE1, GAPDH mRNA expression levels were evaluated by conventional RT-PCR. (**B**) and then protein expression for p-IRE1a, IRE1a, GRP78, FLAG β-actin was determined by Western immunoblot analysis. β-actin served as the standard. (**C**) radiation induced ER-stress was assed via Activation of XBP-1 and CHOP mRNA expression analysis by RT-PCR. GAPDH served as the standard.
